# Contextuality Analysis of the Double Slit Experiment (with a Glimpse into Three Slits)

**DOI:** 10.3390/e20040278

**Published:** 2018-04-12

**Authors:** Ehtibar N. Dzhafarov, Janne V. Kujala

**Affiliations:** 1 Department of Psychological Sciences, Purdue University, West Lafayette, IN 47907, USA; 2 Department of Mathematical Information Technology, University of Jyväskylä, FI-40014 Jyväskylä, Finland

**Keywords:** context-dependence, contextuality, direct influences, double-slit, inconsistent connectedness, signaling, triple-slit

## Abstract

The Contextuality-by-Default theory is illustrated on contextuality analysis of the idealized double-slit experiment. The experiment is described by a system of contextually labeled binary random variables each of which answers the question: Has the particle hit the detector, having passed through a given slit (left or right) in a given state (open or closed)? This system of random variables is a cyclic system of rank 4, formally the same as the system describing the Einsten-Podolsky-Rosen-Bell paradigm with signaling. Unlike the latter, however, the system describing the double-slit experiment is always noncontextual, i.e., the context-dependence in it is entirely explainable in terms of direct influences of contexts (closed-open arrangements of the slits) upon the marginal distributions of the random variables involved. The analysis presented is entirely within the framework of abstract classical probability theory (with contextually labeled random variables). The only physical constraint used in the analysis is that a particle cannot pass through a closed slit. The noncontextuality of the double-slit system does not generalize to systems describing experiments with more than two slits: in an abstract triple-slit system, almost any set of observable detection probabilities is compatible with both a contextual scenario and a noncontextual scenario of the particle passing though various combinations of open and closed slits (although the issue of physical realizability of these scenarios remains open).

## 1. Introduction

**Figure 1:**
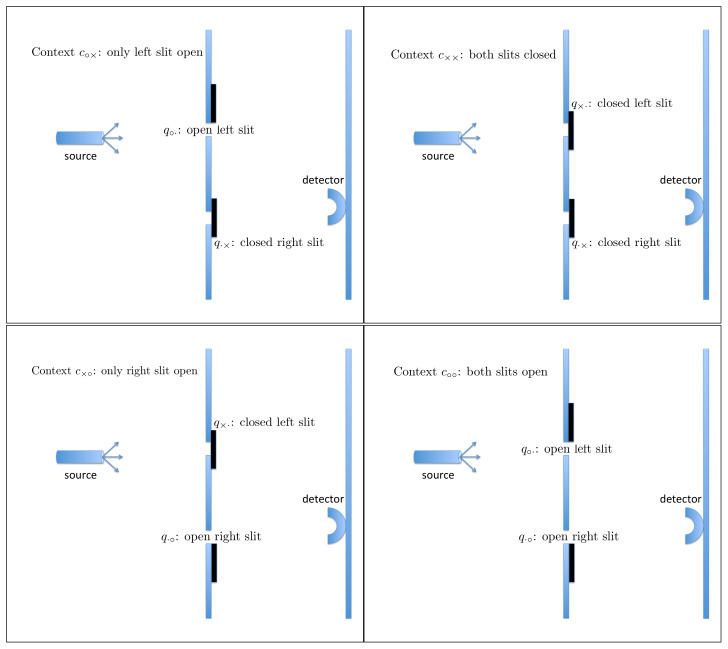
Idealized double slit experiment. The source shoots a single particle that may or may not pass through the slits cut in the intermediate plate, and may or may not be recorded by the detector placed on the screen behind the plate. Each of the slits can be open or closed, and each of the four closed-open arrangements of the two slits forms a context. Each slit (left or right) in each state (open or closed) forms a content, formally treated as the property of the physical system measured by a random variable in a given context.

This note is an illustration of the workings of the Contextuality-by-Default (CbD) theory [1,2,3,4] on the classical double-slit experiment. Specifically, we consider the single-particle version of this experiment, schematically depicted in Figure 1, and represent it by a system of binary random variables $R_{q}^{c}$ answering the question:

**Q1:**  In context c, has the particle emitted by the source hit the detector, having passed through slit *q*?

**Figure 2:**
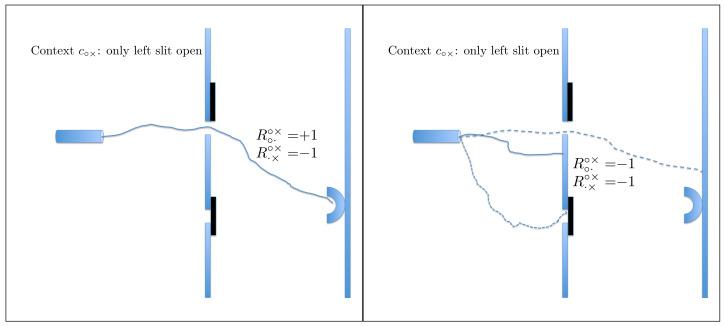
The only physical constraint adopted in our analysis is that a particle can only hit the detector if it has passed through an open slit. The random variables in context $c_{\circ \times }$ are $R_{\circ \cdot }^{\circ \times }$ (has the particle passed through the open left slit and hit the detector?) and $R_{\cdot \times }^{\circ \times }$ (has the particle passed through the closed right slit and hit the detector?). The physical constraint we impose implies that $R_{\cdot \times }^{\circ \times }=-1$ with probability 1, which, in turn, implies that in context $c_{\circ \times }$ the only outcomes that can have nonzero probabilities are as shown in the two panels. The solid irregular curve shows a possible “trajectory” of a single particle, and dashed lines show other possible “trajectories”.

**Figure 3:**
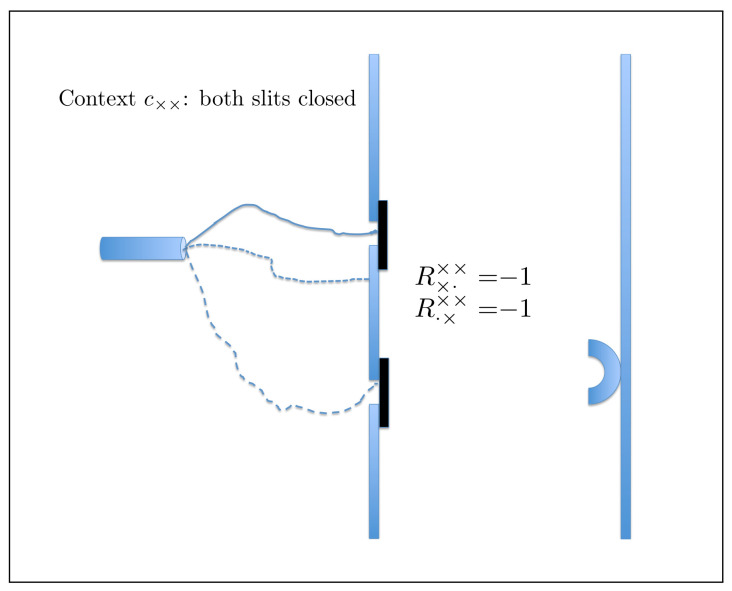
The two random variables in context $c_{\times \times }$ can only attain the values −1. The situation shown therefore occurs with probability 1.

**Figure 4:**
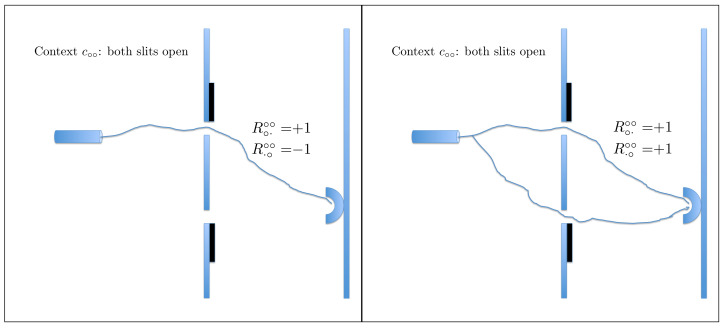
In context $c_{\circ \circ }$, a particle can hit the detector having passed through one of the open slits (**left panel**), but we also allow for the possibility that it passes through both slits (**right panel**), or passes through them several times (not shown). The reason for this is that the outcome of the contextuality analysis does not depend on the probability of the situation in the right panel.

Here, *q* denotes a particular slit, left or right, and whether it is open or closed, whereas *c* denotes the variable part of the experimental set-up: which of the two slits is open and which is closed. The answer to the question Q1 is Yes ($R_{q}^{c}=+1)$ if the conjunction of the following two events occurs: the particle passed through *q*, and the particle hit the detector. If this has not happened, $R_{q}^{c}=-1$. For instance, if $c=c_{\circ \times }$ (the left slit is open, the right one is closed) and $q=q_{\cdot \times }$ (indicating the closed right slit), then $R_{q_{\cdot \times }}^{c_{\circ \times }}=+1$ means that the particle passes through the closed right slit and hits the detector. The probability of this happening is, of course, zero. It is in fact the only physical assumption used in our analysis: that it is impossible for a particle to pass through a closed slit. This can be complemented by the statement (we choose not to consider it as a separate assumption) that it is meaningful to speak of a particle passing or not passing through an open slit. We assume nothing else about possible trajectories, and do not even commit to any specific meaning of the term “trajectory” (the graphical illustrations in Figure 2, Figure 3 and Figure 4 being merely visual aids). We allow the particle to pass through more than one slit at a time, any number of times and in any succession or simultaneously, before hitting the detector or missing it.

We do not set detectors at the slits (which would, as is well known, dramatically change and constrain possible outcomes of the experiment). The only recordable event in our analysis is whether at the end of the experiment the detector placed in the receiving plane has been hit or missed. For any probability of this happening, we therefore have to consider all possible scenarios of whether the particle has passed through this or that of the open slits. This might give rise to the objection that our random variables do not represent any measurements factually performed, whereas, in the traditional contextuality analysis, e.g., in the Kochen–Specker paradigm [5], the random variables always represent results of measurements. This objection would have a merit if the contextuality analysis of the double-slit experiment represented by our random variables $R_{q}^{c}$ led to different conclusions depending on what unobservable scenarios are considered, and if there were no ways of determining, at least in principle, comparative plausibility of different scenarios. We will see, however, that the double-slit system in our analysis turns out to be always noncontextual, making the question of physical plausibility moot. The objection that the events like “the particle passed (did not pass) through this slit” simply do not exist unless measured would be a philosophical disagreement, discussing which here would be out of place. It can be mentioned, however, that the very meaningfulness of closing and opening slits in this experiment is contingent upon one’s believing that something related to the emitted particle somehow passes or fails to pass through these slits. It is not unreasonable therefore to ascribe physical meaning to our random variables, even if not measured.

In Section 5, we show that, with the triple-slit experiment, the situation is different: there, for any nonzero probabilities of the particle eventually hitting the detector in different contexts, one can construct both a contextual scenario and a noncontextual scenario of a particle passing through this or that of the open slits. Because of this, in the absence of physical considerations constraining these scenarios, we consider this result as only “a glimpse” into the triple-slit system, subject to further speculations if not testing.

Contextuality or noncontextuality is a property of a system of random variables representing an empirical situation rather than of the empirical situation itself. Our contextuality analysis pertains to our specific choice of the random variables $R_{q}^{c}$, and it seems it has not been explored previously. There are, however, other possible representations of the double and triple-slit experiments by systems of random variables, and one of them, unrelated to ours, has been considered and will be mentioned in the concluding section.

## 2. Preliminaries: Contextuality-by-Default Approach

The departure point of CbD analysis is representing an empirical situation as a *content-context system* of random variables. This is a set of random variables $R_{q}^{c}$, each of which is labeled by its *content* *q*, which means, roughly, that which the random variable “measures” or “responds to”, and its *context* *c*, the circumstances under which this measurement is made, including but not limited to other contents measured together with a given one. By construction, random variables sharing a context, $R_{q_{1}}^{c},\ldots ,R_{q_{k}}^{c}$, always have a uniquely defined *joint distribution* (they are measured “together”), while any two random variables in different (hence mutually exclusive) contexts, $R_{q}^{c}$ and $R_{q^{\prime }}^{c^{\prime }}$, are *stochastically unrelated*. In particular, in CbD, random variables in different contexts can never be the same. (This allows one to avoid the logical problem one encounters in traditional treatments of contextuality, where the sets of random variables in different contexts have nonempty intersections. The problem arises from two facts: (1) any contextuality analysis aims at establishing the existence or non-existence of certain joint distributions, understood in the classical (Kolmogorovian) sense; (2) in classical probability theory the relation of being jointly distributed is transitive. The conjunction of these two facts makes internally contradictory any claim that, say, a joint distribution of A,B,C does not exist while the pairs A,B and B,C possess joint distributions. For detailed discussion, see [2].)

A *(probabilisitic) coupling* of the system of random variables is a set of jointly distributed random variables $S_{q}^{c}$, in a one-to-one correspondence with $R_{q}^{c}$, such that the joint distribution of any subset of context-sharing $S_{q_{1}}^{c},\ldots ,S_{q_{k}}^{c}$ is the same as that of $R_{q_{1}}^{c},\ldots ,R_{q_{k}}^{c}$. In the traditional analysis of contextuality, the system of random variables $R_{q}^{c}$ is assumed to be *consistently connected*, which means that any two random variables sharing a content, $R_{q}^{c}$ and $R_{q}^{c^{\prime }}$, are identically distributed (while being distinct and stochastically unrelated). The condition of consistent connectedness is known in physics under the names of “no-disturbance”, “no-signaling”, etc. [6,7]. The traditional definition of a *noncontextual system* of random variables $R_{q}^{c}$, formulated in the language of CbD, is that this is a system that has a coupling in which $S_{q}^{c}=S_{q}^{c^{\prime }}$ holds with probability 1 for any two content-sharing random variables $R_{q}^{c}$ and $R_{q}^{c^{\prime }}$. Such a coupling need not exist, and if it does not, the system is *contextual*.

The problem with this definition (and the main motivation behind CbD, besides the need for reconciling contextuality with rigorous probability theory), is that any *inconsistently connected* system of random variables (one in which the distributions of $R_{q}^{c}$ and $R_{q}^{c^{\prime }}$ may differ) is then “automatically” rendered contextual or else placed outside the sphere of applicability of the notion of (non) contextuality. Both these ways of treating inconsistent connectedness, while logically valid, trivialize and severely restrict contextuality analysis. Consistent connectedness is often violated in quantum physics, and it is virtually nonexistent in non-physical applications. Thus, in [1], we re-analyze an experiment [8] exhibiting inconsistent connectedness in the Klyachko–Can–Binicioğlu–Shumvosky paradigm [9]. In the Bohm–Aharonov version of the Einstein–Podolsky–Rosen (EPR) entanglement paradigm [10], famously investigated by Bell and others [11,12,13,14], consistent connectedness is theoretically ensured by space-like separation of the entangled particles. However, in real experiments, inconsistency is often present due to systematic design biases [15]. The two particles may also be time-like separated in some experiments, in which case inconsistent connectedness may be due to factual signaling between the particles [16]. In the Leggett–Garg paradigm [17], later measurements may very well be directly affected by the previous settings (“signaling in time”, [18,19,20,21]), and Bacciagaluppi systematically investigated the ensuing inconsistent connectedness using the CbD approach [22,23]. In behavioral applications, there were several attempts to demonstrate contextuality analogous to the EPR–Bell or Leggett–Garg systems, all these attempts being frustrated by the ubiquity of inconsistent connectedness in behavioral systems (for detailed analysis, see [24,25,26]).

Intuitively, inconsistent connectedness is a manifestation of direct causal action of experimental set-up upon the variables measured in it (hence the terminology of “disturbance”, “invasiveness”, etc.). Contextuality, by contrast, is of a correlational, non-causal nature: even if $R_{q}^{c}$ and $R_{q}^{c^{\prime }}$ are identically distributed, their correlations with other random variables in the respective contexts make it impossible to map them into two always-equal $S_{q}^{c}$ and $S_{q}^{c^{\prime }}$ within a coupling. In other words, the difference in the *identities* of the two random variables cannot be explained by the difference of their distributions (in this case, no difference). A random variable is *identified* as a measurable function from a probability space into a measurable space. *Distribution* (the measure induced by this mapping in the codomain space) is only one aspect of the random variable’s identity. It seems reasonable therefore to extend the definition of contextuality to allow (non)contextuality and (in)consistent connectedness to coexist in all four possible combinations. In CbD, this is achieved by considering the maximal possible probability with which jointly distributed $S_{q}^{c}$ and $S_{q}^{c^{\prime }}$ (having the same individual distributions as $R_{q}^{c}$ and $R_{q}^{c^{\prime }}$, respectively) can be equal to each other. This probability equals 1 if the two distributions are the same, and if they are not, it is viewed as a measure of the difference between them. The question of contextuality then is translated into whether this difference in distributions is sufficient to account for the difference between the random variables’ identities:

**Q2:**  Given (generally different) distributions of $R_{q}^{c}$ and $R_{q}^{c^{\prime }}$, do their correlations with other random variables in their respective contexts make it possible to map them into jointly distributed $S_{q}^{c}$ and $S_{q}^{c^{\prime }}$ (within a coupling of the system containing $R_{q}^{c}$ and $R_{q}^{c^{\prime }}$) that are equal to each other with the maximal possible probability?

The main idea underlying CbD is that, if this question is answered in the affirmative for every pair of content-sharing random variables, the system is noncontextual. Otherwise, it is contextual. An important initial step in the analysis is that each random variable in the system is to be dichotomized, replaced by a set of binary variables, for reasons discussed in [2,3,4] (In a nutshell, a system of random variables amenable to contextuality analysis should satisfy certain desiderata, such as uniqueness of the coupling for any set of content-sharing random variables, and the preservation of noncontextuality under deletions and coarse-graining of the random variables). We skip this discussion, as the system to be dealt with in this paper consists of random variables that are already binary. As this system turns out to be noncontextual, we also skip the otherwise important issue of measuring the *degree of contextuality* in systems found to be contextual [27,28].

## 3. The Content-Context Representation of the Double-Slit Experiment

The content-context system of the random variables we have chosen to represent the double-slit experiment is

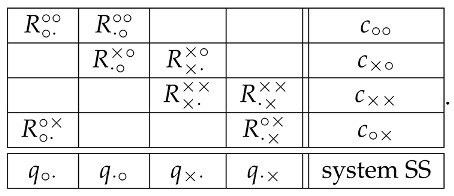
(1)

The superscripts of the random variables show their contexts, the left (right) symbol indicating whether the left (right) slit is open (∘) or closed (×). The subscript of each random variable shows which of these two slits is being “measured” by this random variable, i.e., about which slit we ask the Question Q1: e.g., ∘· in $R_{\circ \cdot }^{\circ \times }$ shows that the question is being asked about the left open slit (when the right one is closed). The random variables can be arranged in the following cyclic structure:

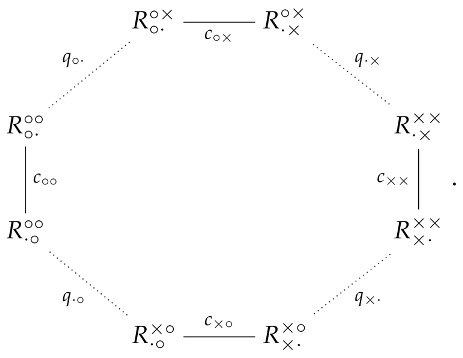
(2)

According to CbD, the random variables sharing a context, i.e., those in the same row of matrix (1) and connected by solid lines in the diagram (2), are jointly distributed. Let us present these distributions with references to the corresponding graphical illustrations:

(3) $$\left(\mathrm{see}\;\mathrm{Figure}\;2\right)\;\begin{array}{cccc} \mathrm{context}\;c_{\circ \times } & R_{\cdot \times }^{\circ \times }=+1 & R_{\cdot \times }^{\circ \times }=-1\\ R_{\circ \cdot }^{\circ \times }=+1 & 0 & p & p\\ R_{\circ \cdot }^{\circ \times }=-1 & 0 & 1-p & 1-p\\ & 0 & 1 \end{array},$$

(4) $$\left(\mathrm{see}\;\mathrm{Figure}\;3\right)\;\begin{array}{cccc} \mathrm{context}\;c_{\times \times } & R_{\cdot \times }^{\times \times }=+1 & R_{\cdot \times }^{\times \times }=-1\\ R_{\times \cdot }^{\times \times }=+1 & 0 & 0 & 0\\ R_{\times \cdot }^{\times \times }=-1 & 0 & 1 & 1\\ & 0 & 1 \end{array},$$

(5) $$\begin{array}{c} (\mathrm{same}\;\mathrm{as}\;\mathrm{Figure}\;2\\ \;\mathrm{with}\;\mathrm{left}\;\mathrm{and}\\ \;\mathrm{right}\;\mathrm{reversed}) \end{array},\;\begin{array}{cccc} \mathrm{context}\;c_{\times \circ } & R_{\cdot \circ }^{\times \circ }=+1 & R_{\cdot \circ }^{\times \circ }=-1\\ R_{\times \cdot }^{\times \circ }=+1 & 0 & 0 & 0\\ R_{\times \cdot }^{\times \circ }=-1 & q & 1-q & 1\\ & 0 & 1 \end{array},$$

(6) $$\left(\mathrm{see}\;\mathrm{Figure}\;4\right)\;\begin{array}{cccc} \mathrm{context}\;c_{\circ \circ } & R_{\cdot \circ }^{\circ \circ }=+1 & R_{\cdot \circ }^{\circ \circ }=-1\\ R_{\circ \cdot }^{\circ \circ }=+1 & r^{\prime } & p^{\prime } & r^{\prime }+p^{\prime }\\ R_{\circ \cdot }^{\circ \circ }=-1 & q^{\prime } & 1-p^{\prime }-q^{\prime }-r^{\prime } & 1-r^{\prime }-p^{\prime }\\ & r^{\prime }+q^{\prime } & 1-r^{\prime }-q^{\prime } \end{array}.$$

In each of these distributions, the only observable probability (i.e., one that can be estimated from empirical data) is the nondetection probability shown in boldface. The rest of the probabilities represent the scenarios of the particle passing through the open slits in all imaginable ways.

## 4. Contextuality Analysis of the Double-Slit Experiment

According to CbD, the system shown in (1) and (2) is noncontextual if and only if one can find eight jointly distributed random variables

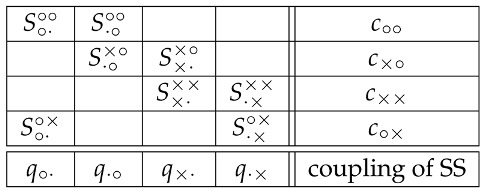
(7) in one-to-one correspondence with the elements of (1), with the following properties:

1. The variables $S_{q}^{c}$ form a coupling of the system (1). This means that in each row of (7) the random variables have the same joint distribution as in the corresponding row of (1).
2. This coupling is *multimaximally connected*. This means that in each column of (7) the two random variables have the joint distribution in which they are equal to each other with the maximal possible probability. This maximal probability is constrained by the individual distributions of the two random variables, coinciding with those of the corresponding variables in (1). (If the system contained more than two variables in a column, this maximality requirement would be applied to every pair of them. With all variables binary, this requirement can always be satisfied and in precisely one possible way [2,3]).

The first requirement is simple: all probabilities shown in (3)–(6) remain unchanged if one replaces each $R_{q}^{c}$ in them with the corresponding $S_{q}^{c}$. To understand the second requirement, consider, e.g., the first column in (7). The probability of $S_{\circ \cdot }^{\circ \circ }=S_{\circ \cdot }^{\circ \times }$ is the sum of $\mathrm{Prob}\;\left[S_{\circ \cdot }^{\circ \circ }=S_{\circ \cdot }^{\circ \times }=1\right]$ and $\mathrm{Prob}\;\left[S_{\circ \cdot }^{\circ \circ }=S_{\circ \cdot }^{\circ \times }=-1\right]$, and their maximal possible values are

(8) $$\begin{array}{cc} \mathrm{Prob}\;[S_{\circ \cdot }^{\circ \circ }=1,S_{\circ \cdot }^{\circ \times }=1] & =\min\;(\mathrm{Prob}\;[S_{\circ \cdot }^{\circ \circ }=1],\mathrm{Prob}\;[S_{\circ \cdot }^{\circ \times }=1]),\\ \mathrm{Prob}\;[S_{\circ \cdot }^{\circ \circ }=-1,S_{\circ \cdot }^{\circ \times }=-1] & =\min\;(\mathrm{Prob}\;[S_{\circ \cdot }^{\circ \circ }=-1],\mathrm{Prob}\;[S_{\circ \cdot }^{\circ \times }=-1]). \end{array}$$

This determines the joint probability of $\left(S_{\circ \cdot }^{\circ \circ },S_{\circ \cdot }^{\circ \times }\right)$ uniquely. Using the probabilities shown in (3) and (6),

(9) $$\begin{array}{cccc} \mathrm{content}\;q_{\circ \cdot } & S_{\circ \cdot }^{\circ \times }=+1 & S_{\circ \cdot }^{\circ \times }=-1\\ S_{\circ \cdot }^{\circ \circ }=+1 & \min\left(p,r^{\prime }+p^{\prime }\right) & r^{\prime }+p^{\prime }-\min\left(p,r^{\prime }+p^{\prime }\right) & r^{\prime }+p^{\prime }\\ S_{\circ \cdot }^{\circ \circ }=-1 & p-\min\left(p,r^{\prime }+p^{\prime }\right) & \min\left(1-p,1-r^{\prime }-p^{\prime }\right) & 1-r^{\prime }-p^{\prime }\\ & p & 1-p \end{array}.$$

The joint distributions for the remaining three contents (columns) of (7) are computed similarly.

We see therefore that, in the hypothetical coupling (7), the distributions in each row and in each column are uniquely specified. The question of whether the system (1) is (non)contextual becomes the question of whether these row-wise and column-wise distributions in (7) are mutually compatible, i.e., whether there is a joint distribution of all eight random variables in (7) with these row-wise and column-wise distributions as its marginals. Our system of random variables (1) and (2) is a cyclic system of rank 4 [27], also used to describe the EPR–Bell experiment with spin-1/2 particles [12,13,14]. One can therefore answer the question about compatibility by using the criterion of (non)contextuality of a cyclic system derived in [29].

In general, a cyclic system of rank n≥2 consists of 2n binary random variables arranged so that each context $c_{i}$ (i=1,…,n) is defined by two contents $q_{i},q_{i⊕1}$ measured together, and each content $q_{i⊕1}$ enters in two contexts $c_{i},c_{i⊕1}$ (where i⊕1 is simply i+1 except for n⊕1=1). This system is noncontextual (i.e., it has a multimaximally connected coupling) if and only if

(10) $$\max_{\left\{\lambda_{1},\ldots ,\lambda_{n}\right\}\in \Lambda_{n}}\sum_{i=1}^{n}\lambda_{i}\langle R_{i}^{i}R_{i⊕1}^{i}\rangle \leq n-2+\sum_{i=1}^{n}\left|\langle R_{i⊕1}^{i}\rangle -\langle R_{i⊕1}^{i⊕1}\rangle \right|,$$

where $\Lambda_{n}$ denote the set of *n*-tuples $\left\{\lambda_{1},\ldots ,\lambda_{n}\right\}$ such that $\lambda_{i}\in \left\{-1,+1\right\}$ and $\prod_{i=1}^{n}\lambda_{i}=-1$ (i.e., the number of the minus signs in the left-hand side sum is odd). If the system is consistently connected, the sum of $\left|\langle R_{i⊕1}^{i}\rangle -\langle R_{i⊕1}^{i⊕1}\rangle \right|$ in the right-hand side disappears, and the criterion coincides with the one derived (in a very different way) in [30].

By simple if tedious algebra, the expected values entering (10) can be computed for n=4 using (3)–(6), and the result is that (10) is satisfied irrespective of the probability values in (3)–(6). The double-slit experiment represented by our random variables (1) and (2) is always noncontextual.

There is, however, a much simpler way of establishing this noncontextuality. In the matrix (1), the random variables with contents $q_{\times \cdot }$ and $q_{\cdot \times }$ are deterministic (equal to −1 with probability 1). As shown in [4], adding or deleting a deterministic quantity to/from a system of random variables does not change its contextuality or noncontextuality. (In fact, the statement is stronger: the system’s *degree of contextuality* does not change. We do not discuss this notion here.) The system therefore is equivalent (with respect to its contextuality) to

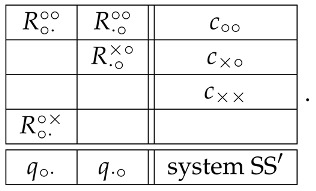
(11)

It is also clear that deleting a context containing just one or no random variables does not change the system’s contextuality or noncontextuality. (Again, the statement is stronger: the system’s degree of contextuality does not change.) The system therefore can be replaced with

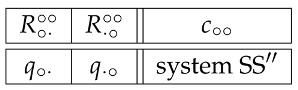
(12) whose noncontextuality is trivially apparent.

## 5. A Glimpse into the Triple-Slit System

The noncontextuality of the double-slit system does not depend on whether it is physically realizable: it holds for any system (1). The situation with systems with three or more slits is different. Consider the triple-slit system

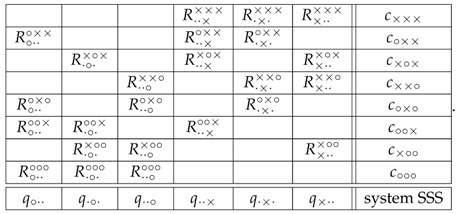
(13)

Using the same shortcut reasoning as with the system (1), i.e., deleting the columns with deterministic variables and the rows with no more than one random variable, this triple-slit system is equivalent (with respect to its contextuality) to

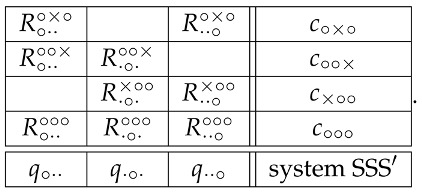
(14)

Let the nondetection probabilities (the only observable ones) in these four contexts be denoted

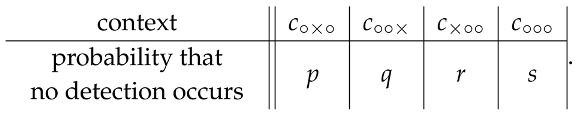
(15)

One can always find a noncontextual scenario for these probabilities, e.g., the following one, in which all but one random variable in each context are deterministic: in context $c_{\circ \circ \circ }$,

(16) $$\mathrm{Prob}\;\left[\begin{array}{c} R_{\circ \cdot \cdot }^{\circ \circ \circ }=i\\ R_{\cdot \circ \cdot }^{\circ \circ \circ }=j\\ R_{\cdot \cdot \circ }^{\circ \circ \circ }=k \end{array}\right]=\left\{\begin{array}{ccc} s, & \mathrm{if} & \left(i,j,k\right)=\left(-1,-1,-1\right),\\ 1-s, & \mathrm{if} & \left(i,j,k\right)=\left(+1,-1,-1\right),\\ 0, & \mathrm{if} & \mathrm{otherwise}, \end{array}\right.$$

and, in the three remaining contexts,

(17) $$\begin{array}{c} \begin{array}{cccc} c_{\circ \times \circ } & R_{\cdot \cdot \circ }^{\circ \times \circ }=+1 & R_{\cdot \cdot \circ }^{\circ \times \circ }=-1\\ R_{\circ \cdot \cdot }^{\circ \times \circ }=+1 & 0 & 1-p & 1-p\\ R_{\circ \cdot \cdot }^{\circ \times \circ }=-1 & 0 & p & p\\ & 0 & 1 \end{array},\\ \begin{array}{cccc} c_{\circ \circ \times } & R_{\cdot \circ \cdot }^{\circ \circ \times }=+1 & R_{\cdot \circ \cdot }^{\circ \circ \times }=-1\\ R_{\circ \cdot \cdot }^{\circ \circ \times }=+1 & 0 & 0 & 0\\ R_{\circ \cdot \cdot }^{\circ \circ \times }=-1 & 1-q & q & 1\\ & 1-q & q \end{array},\\ \begin{array}{cccc} c_{\times \circ \circ } & R_{\cdot \cdot \circ }^{\times \circ \circ }=+1 & R_{\cdot \cdot \circ }^{\times \circ \circ }=-1\\ R_{\cdot \circ \cdot }^{\times \circ \circ }=+1 & 0 & 1-r & 1-r\\ R_{\cdot \circ \cdot }^{\times \circ \circ }=-1 & 0 & r & r\\ & 0 & 1 \end{array} \end{array}.$$

The nondetection probabilities (15) are also compatible with contextual scenarios, with some exceptions, e.g., if any three of them equal 1. Not to deal with special cases, we construct a contextual scenario under the additional assumption that s<1 and p<1 (where *p* can be replaced with *q* or *r*). Choose a probability $t>\max\left(s,p\right)$ and put

(18) $$\mathrm{Prob}\;\left[\begin{array}{c} R_{\circ \cdot \cdot }^{\circ \circ \circ }=i\\ R_{\cdot \circ \cdot }^{\circ \circ \circ }=j\\ R_{\cdot \cdot \circ }^{\circ \circ \circ }=k \end{array}\right]=\left\{\begin{array}{ccc} s, & \mathrm{if} & \left(i,j,k\right)=\left(-1,-1,-1\right),\\ t-s, & \mathrm{if} & \left(i,j,k\right)=\left(-1,+1,-1\right),\\ 1-t, & \mathrm{if} & \left(i,j,k\right)=\left(+1,+1,+1\right),\\ 0, & \mathrm{if} & \mathrm{otherwise}. \end{array}\right.$$

Consider the subsystem

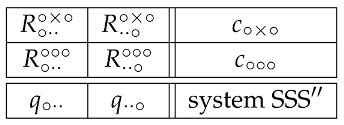
(19) of the system (14). Define the two row-wise distributions as

(20) $$\begin{array}{c} \begin{array}{cccc} c_{\circ \times \circ } & R_{\cdot \cdot \circ }^{\circ \times \circ }=+1 & R_{\cdot \cdot \circ }^{\circ \times \circ }=-1\\ R_{\circ \cdot \cdot }^{\circ \times \circ }=+1 & 1-2t+p & t-p & 1-t\\ R_{\circ \cdot \cdot }^{\circ \times \circ }=-1 & t-p & p & t\\ & 1-t & t \end{array},\\ \begin{array}{cccc} c_{\circ \circ \circ } & R_{\cdot \cdot \circ }^{\circ \circ \circ }=+1 & R_{\cdot \cdot \circ }^{\circ \circ \circ }=-1\\ R_{\circ \cdot \cdot }^{\circ \circ \circ }=+1 & 1-t & 0 & 1-t\\ R_{\circ \cdot \cdot }^{\circ \circ \circ }=-1 & 0 & t & t\\ & 1-t & t \end{array} \end{array}.$$

This describes a consistently connected cyclic system of rank 2. The contextuality of this subsystem (hence also the contextuality of the entire system) can be verified by applying to it the criterion (10) with n=2, or simply observing that this system can be noncontextual only if t=p.

## 6. Conclusions

We have established that the system of random variables describing the double-slit experiment (in terms of which open slits the particle passes through before hitting or missing the detector) is noncontextual for all possible scenarios. For experiments involving more than two slits, the systems describing them can be contextual. In fact, excluding some special cases, every set of observable (in the statistical sense) detection probabilities in this case allows for a contextual scenario and a noncontextual scenario. The interpretation of the noncontextuality of the double-slit system is that all context-dependence in this system is due to direct influences exerted by the state of a slit (open or closed) upon the probabilities with which a particle passes through the other slit and hits the detector. These direct influences are manifested in the differences in the distributions of random variables sharing a content (tied to the same open slit). By contrast, one can construct triple-slit systems (on paper, their physical realizability is open to investigation), in which the difference in the identity of random variables tied to a given slit under different (open-closed) arrangements of other slits cannot be accounted by the difference in the distributions of these random variables alone: we have a “pure contextuality” here, on top of any possible direct influences. Physical mechanisms of direct influences play no role in our analysis. With the exception of the prohibition for a particle to pass through a closed slit, the analysis involves no physical assumptions whatever.

As mentioned in the introductory part of the paper, contextuality analysis characterizes a set of random variables rather than an empirical situation that, while it can be described by this set of random variables, allows for other descriptions. Our analysis pertains to a particular choice of random variables, tying each of them to a particular slit (left or right) in a particular state (open or closed). Each context in our analysis involves two random variables in no particular chronological relation to each other. Kofler and Brukner [18] explored another way of looking at the double-slit experiment (more precisely, at its simplified version provided by a Mach–Zehnder interferometer). The contents there correspond to three chronological stages, $t_{1}$ (the stage preceding the first beam split), $t_{2}$ (between the first and the second splits), and $t_{3}$ (following the second split). With each of these stages, one associates a binary random variable whose values corresponds to the choice of one two possible paths. The measurements are assumed to be made in pairs, $\left(t_{1},t_{2}\right)$, $\left(t_{1},t_{3}\right)$, and $\left(t_{2},t_{3}\right)$, forming three contexts. In the CbD language, this creates six contextually labeled random variables forming a cyclic system of rank 3, essentially the same as one used to describe the Leggett–Garg experiment [17]. Kofler and Brukner discuss contextuality of this system for the case when it is consistently connected. Mansfield, in an unpublished conference presentation [31], also discussed a cyclic-3 representation for the double-slit experiment, but, in a more general version, allowing for “signaling in time”. We see no obvious relations between these analyses and ours, and they are only mentioned here for completeness.

Richard Feynman is often cited as asserting that the double-slit experiment is incompatible with classical probability. He characterized the interference pattern as “the discovery that in nature the laws of combining probabilities were not those of the classical probability theory of Laplace” ([32] p. 533), and he said that it is “a phenomenon which is impossible, *absolutely* impossible, to explain in any classical way” ([33], Section 37-1). Although one can think of alternative interpretations for these quotes and find other quotes seemingly saying something else, this interpretation is widely accepted (see, e.g., [34,35,36,37]). Our analysis contradicts this interpretation, whether historically correct or not, as CbD is squarely an application of classical (Kolmogorovian) probability theory. Feynman’s claim (or alleged claim) has been challenged by others as well, and all of these challenges were using some form of contextual labeling of the random variables involved. Thus, Ballantine [36] and Khrennikov [38,39,40] treat the probabilities p,q,… in matrices like our (3)–(6) as *conditional probabilities*, using the contexts as conditioning random events. Even closer to CbD, Khrennikov [41] treats p,q,… as “contextual probabilities”, with $c_{\circ \times }$, $c_{\times \times }$, being essentially labels rather than conditioning events. In all these and similar treatments, the conditional labeling is used to show that the classical probabilistic formulas claimed to be violated by quantum-mechanical phenomena simply do not apply. For instance, the additivity of probabilities of disjoint events, thought by Feynman to be violated by the double-slit experiment, does not apply because the union of the disjoint events and the events themselves are conditioned (or “contextualized”) by different contexts. In CbD, this is definitely true, but this is only a departure point for subsequent contextuality analysis [42].
